# Vitamin D protects intestines from liver cirrhosis-induced inflammation and oxidative stress by inhibiting the TLR4/MyD88/NF-κB signaling pathway

**DOI:** 10.1515/med-2023-0714

**Published:** 2023-05-29

**Authors:** Mei Luo, Yuanhong Xu, Jike Li, Dongxia Luo, Li Zhu, Yanxi Wu, Xiaodong Liu, Pengfei Wu

**Affiliations:** Infectious Disease Laboratory, Chengdu Public Health Clinical Center, Chengdu, 610061, China; Clinical Laboratory, Chengdu Public Health Clinical Center, Chengdu, 610061, China; Hepatology Clinic, Chengdu Public Health Clinical Center, Chengdu, 610061, China

**Keywords:** liver cirrhosis, vitamin D, intestinal injury, α-defensin 5, oxidative stress

## Abstract

Liver cirrhosis affects the structures and physiological functions of the intestine. Our previous study revealed that liver injury inhibited 25-hydroxylation of vitamin D (25(OH)-VD). The aim of this study was to investigate the roles and mechanisms of vitamin D in liver cirrhosis-induced intestinal injury. The rat liver cirrhosis model was established through the administration of carbon tetrachloride (CCl_4_) for 8 weeks. Hematoxylin–eosin staining was performed to unveil the intestinal injury induced by liver cirrhosis. Enzyme-linked immunosorbent and reverse transcription PCR (RT-PCR) analysis were used to determine the levels of 25(OH)-VD, vitamin D receptor, Cytochrome P450 24A1 (CYP24A1), and α-defensin 5 (DEFA5) in rat and human serum of liver cirrhosis. Furthermore, liver cirrhosis rats were treated with low-dose (500 IU/kg) and high-dose (2,000 IU/kg) vitamin D intraperitoneally. The expression levels of TLR4/MyD88/NF-κB signaling pathway were evaluated by RT-PCR and Western blot. In conclusion, we determined the deficiency of vitamin D and down-regulation of DEFA5 and intestinal damage induced by liver cirrhosis. Moreover, vitamin D effectively inhibited liver cirrhosis-induced intestinal inflammation and oxidative stress through the TLR4/MyD88/NF-κB pathway. Vitamin D might be a promising therapeutic strategy for future treatment of liver-induced intestinal injury.

## 1 Introduction

Cirrhosis is an advanced stage of liver fibrosis with the development of regenerative nodules of liver parenchyma separated by fibrotic septa with accompanying ascites, renal failure, hepatic encephalopathy, and variceal bleeding [1]. Besides, liver cirrhosis is divided into compensated and decompensated cirrhosis [2]. Patients with compensated cirrhosis may remain free of severe complications for several years, but decompensated cirrhosis has short overall survival [3]. Despite the advances in liver cirrhosis therapeutics, the treatments for cirrhosis still remain challenging. Liver transplantation is a primary intervention of cirrhosis, but the shortage of available donor organs severely restricted the applicability [4]. It has been reported that liver function is closely associated with intestinal barrier and microenvironment [5,6]. The liver–gut axis refers to the bidirectional relationship between the intestinal and the liver. The portal vein directly connects the liver to the intestinal and regulates the transfer of nutrients and microbial components along the liver–gut axis [7,8]. The damage of the intestinal barrier could be found in the animal liver failure model [6], revealing that liver failure may further facilitate the injury of intestinal biological functions.

Vitamin D is a class of fat-soluble vitamins that act a crucial role on intestinal absorption of calcium, phosphate, and other essential biological components [9]. Vitamin D receptor (VDR) is abundantly expressed in the distal ileum, where the Paneth cells are enriched [10]. Previous researches demonstrate that vitamin D is involved with intestinal immune system [11]. And the vitamin D deficiency could deteriorate the intestinal barrier function and dysbiosis [12]. Findings relating the relationship between the vitamin D and the innate defenses against bacterial pathogens have been reported [13]. 25(OH)VD_3_ could triggered a host-defense response in cattle by up-regulating plenty of β-defensins genes, which suggested that vitamin D acted crucial role in the protection of cattle from bacterial and viral pathogens [13]. In the present study, we found the deficiency of vitamin D and down-regulation of α-defensin 5 (DEFA5) in intestines induced by liver cirrhosis. Therefore, we reasoned that liver cirrhosis inhibited the 25-hydroxylation of vitamin D (25(OH)-VD) in intestines and, thereby, attenuating the secretion of DEFA5 and leading to sustained injury to the intestinal barrier.

Previous reports have demonstrated the protective roles of vitamin D in intestinal structures and biological functions. However, the molecular mechanisms underlying the effects of vitamin D on intestinal injury in cirrhosis remain unclear. TLR4 has been involved in the process of intestinal injury, inflammation, and oxidative stress [14]. Previous report indicated that the absence of TLR4 or TLR4-induced signaling attenuated local mucosal damage with significantly decreased cytokine and eicosanoid secretion including PGE2 production, thus promoting intestinal ischemia/reperfusion-induced damage [15]. Dysregulated intestinal TLR4 activation led to chronic inflammation and intestinal epithelial apoptosis in the setting of necrotizing enterocolitis, which is a key factor of death among preterm infants [16]. In this study, we revealed that vitamin D suppressed liver cirrhosis-induced intestinal inflammation and oxidative stress through the TLR4/MyD88/NF-κB pathway. The treatment of TLR4 agonist (GSK1795091) and ROS inducer (diallyl tetrasulfide) remarkably reversed the vitamin D-enhanced intestinal protection.

## 2 Materials and methods

### 2.1 Animals and clinical blood samples

A total of 30 adult male SD rats (200–220 g, 6 weeks old) were obtained from Shanghai Model Organisms Center. We have successfully constructed the liver cirrhosis model using male rats according to a previous report [17]. The same results are expected in female animals.

**Table 1:** The clinical characteristics

	Liver cirrhosis group	Control group
Age	49.81 ± 1.147, *N* = 74	35.09 ± 1.937, *N* = 35
ALT (U/L)	86.10 ± 16.74, *N* = 69	19.23 ± 2.102, *N* = 35
ALP (U/L)	157.4 ± 17.67, *N* = 69	61.89 ± 3.705, *N* = 35
GGT (U/L)	95.09 ± 18.38, *N* = 69	16.71 ± 1.635, *N* = 35
TB (μmol/L)	63.91 ± 14.90, *N* = 69	10.87 ± 0.7275, *N* = 35
BUN (mmol/L)	5.477 ± 0.6853, *N* = 58	4.485 ± 0.2247, *N* = 35
Crea (μmol/L)	74.24 ± 9.463, *N* = 58	52.44 ± 1.637, *N* = 35
HA (ng/mL)	201.8 ± 22.57, *N* = 57	*N*
LN (ng/mL)	95.09 ± 7.023, *N* = 58	*N*
PCIII (ng/mL)	216.8 ± 41.96, *N* = 58	*N*
IV-col (ng/mL)	157.6 ± 21.95, *N* = 58	*N*


**Ethics approval and consent to participate:** All procedures were approved by the Ethics Committee of Chengdu Public Health Clinical Center (Approval No: PJ-K2020-51-01, Chengdu, China). All of the blood samples were collected after written informed consent and the clinical characteristics are listed in Table 1.

### 2.2 Liver cirrhosis model and vitamin D therapy

Briefly, the rats were treated with CCl_4_ (Cat No. C07315202, Nanjing Reagent, China) by oral gavage at a dose of 2 mL/kg body weight twice a week for 8 weeks to generate a liver cirrhosis model. The cirrhosis rats were randomly divided into five groups (*n* = 5 per group). The low-dose vitamin D (VD-L) and high-dose vitamin D (VD-H) rats were injected daily of vitamin D injection (Zhejiang Xianju Pharmaceutical Co., Ltd, Taizhou, China) intraperitoneally [18,19,20]. Besides, GSK1795091 (HY-111792; MedChemExpress, Princeton, NJ, USA) and diallyl tetrasulfide (Dat, ab143603; Abcam, Shanghai, China) were administered by tail vein injection to induce TLR4 and ROS levels, respectively. All rats were allowed free access to water and standard rat chow. Rats were anesthetized with isoflurane inhalation (induction dose of 5% and maintenance dose of 1.5%) and 3 mL of blood collected from the inferior vena cava in an EDTA tube, which was centrifuged at 1,500 rpm for 10 min to get the serum. After sacrificing the rats, the liver and ileum tissues of every rat were clipped and stored at −80℃ for the follow-up experiments.

Control group: Rats received oral administrations of the same volume of saline.

Cirrhosis group: Rats received CCl_4_ by oral gavage at a dose of 2 mL/kg for 8 weeks.

Cirrhosis + VD-L group: Rats received vitamin D (500 IU/kg) on the fourth week of CCl_4_ treatment.

Cirrhosis + VD-H group: Rats received vitamin D (2,000 IU/kg) on the fourth week of CCl_4_ treatment.

Cirrhosis + VD-H + GSK1795091 group: Rats received vitamin D (2,000 IU/kg) and GSK1795091 (20 mg/kg) on the fourth week of CCl_4_ treatment.

Cirrhosis + VD-H + Dat group: Rats received vitamin D (2,000 IU/kg) and Dat (25 mg/kg) on the fourth week of CCl_4_ treatment.

### 2.3 Liver function and histological evaluation

Liver function was assessed by the serum alanine aminotransferase (ALT), aspartate aminotransferase (AST), alkaline phosphatase (ALP), and albumin (ALB). The blood serum samples were obtained and subjected to analysis using an automatic biochemical analyzer (HITACHI 7600, Japan). Moreover, after being fixed with 10% formalin and embedded with paraffin, the liver tissues were cut into 4 µM slices for hematoxylin and eosin (HE) staining and Masson staining analyses according to the methods described previously to evaluate the development of liver fibrosis [21]. In addition, the ileum tissues of each group were stained with HE to examine the effect of liver cirrhosis on the intestinal morphological structure. The sections were photographed under a light microscope (Olympus BX51, Japan).

### 2.4 Immunohistochemistry

The clinical ileum tissues were collected and fixed with 4% paraformaldehyde. Then, the tissues were dehydrated, cleared, and paraffin-embedded. Four-micrometer paraffin sections were incubated with primary antibodies against ZO-1 (*dilution ratio* 1:1,000, ab221546; Abcam) at 4°C overnight, followed by incubation with goat anti-rabbit horseradish peroxidase (*dilution ratio* 1:50, A0208; Beyotime, Shanghai, China) for 1 h at room temperature. Subsequently, the slides were stained with DAB and counterstained with hematoxylin for 30 s, followed by conventional treatments. Finally, photographs were photographed under observation by a light microscope (Olympus BX51, Japan).

### 2.5 Detection of the levels of 25(OH)VD_3_, VDR, CYP24A1, and DEFA5

Serum 25(OH)VD_3_ levels were analyzed through enzyme immunoassay using the 25-Hydroxy Vitamin D3 ELISA kit (Cat No. LS-F5643; LifeSpan Biosciences, USA). Serum VDR concentrations were detected using Enzyme-linked Immunosorbent Assay Kit (Cat No. MBS022460; MyBioSource, USA). The levels of CYP24A1 were determined by Cytochrome P450 24A1 (CYP24A1) ELISA kit (Cat No. abx508211; Abbexa, USA). The Defensin 5 (Sandwich ELISA) kit (Cat No. LS-F33773; LifeSpan Biosciences) was applied to measure the levels of DEFA5 in human blood samples. The DEFA5 level of rat intestinal tissues was determined using the reverse transcription polymerase chain reaction (RT-PCR) analysis.

### 2.6 Measurement of inflammatory cytokines and oxidative stress

The levels of inflammatory cytokines, including IL-6, CXCL1, IL-1β and TNF-α, in the ileum tissues were determined with ELISA kits (Beyotime). And the catalog numbers were listed as follows: IL-6 (PI335), CXCL1 (PC175), IL-1β (PI303), and TNF-α (PT516). Besides, the oxidative stress markers were detected in the ileum tissues of the rats. Ileum samples were homogenized in lysis buffer containing 50 mM Tris (pH 7.4), 150 mM NaCl, 1% Triton X-100, 1% sodium deoxycholate, and 0.1% SDS, and the supernatants were used for the detection. The malondialdehyde (MDA) content and the activities of catalase (CAT), superoxide dismutase (SOD), and glutathione peroxidase (GSH-Px) were measured using MDA, CAT, SOD, and GSH-Px ELISA kits ((Nanjing Jiancheng Bioengineering Institute, China) according to the manufacturer’s protocol. And the catalog numbers were listed as follows: MDA (A003-1-2), CAT (A007-2-1), SOD (A001-3-2), and GSH-Px (A005-1-2).

### 2.7 Western blot

The ileum tissues were lysed in RIPA buffer (Cat No. P0013C; Beyotime) and the total protein concentrations were detected through a BCA kit (Cat No. P0012S; Beyotime). Immunoblotting was carried out as previously reported. Proteins (50 μg/lane) were separated by 15% sodium dodecyl sulfate polyacrylamide gel electrophoresis gels. After blocking the PVDF membrane (Cat No. IPVH00010; Millipore, USA), they were subsequently incubated overnight with primary antibodies against TLR4 (*dilution ratio* 1:1,000, AF7017; Affinity, China), MyD88 (*dilution ratio* 1:500, AF5192; Affinity), p-NF-kB (*dilution ratio* 1:1,000, ab194726; Abcam), NF-kB (*dilution ratio* 1:1,000, ab16502; Abcam), and GAPDH (*dilution ratio* 1:3,000, AF7021; Affinity). Then, the membranes were incubated with secondary HRP-conjugated antibodies (*dilution ratio* 1:3,000, S0001; Affinity) at room temperature for 2 h. The immunoreactive bands were then analyzed using the Western ECL reagent (Cat No. RPN2106V1, GE Healthcare, UK) according to the protocol of the manufacturer.

### 2.8 RT-PCR analysis

Total RNA was isolated by TRIzol reagent (Cat No. 10296010; Invitrogen, USA) according to the manufacturer’s instructions. cDNA was derived from 1 μg RNA using SuperScript III Reverse Transcriptase (Cat No.18080044; Invitrogen). Quantitative PCR was performed with an SYBR-Green Realtime PCR Master Mix (Cat No. FSK-101; ToyoBo Life Sciences, Japan) in a CFX Real-Time System (Bio-Rad, Germany). The sequences of primers for DEFA5 were forward, ATCCACTCCTGCTCTCCCTC, and reverse, AGAAAGACACAAGGTACACAGAGT. The sequences of primers for TLR4 were forward, ACTGGGTGAGAAACGAGCTG, and reverse, GTCCACAGCAGAAACCCAGA. The primers for MyD88 were forward, GTTTGTGCTTCCGGGAACAC, and reverse, GCAAAGAGGCCTCCATTCCT. The primers for NF-kB were forward, ACGGTGGGATTGCATTCCAT, and reverse, GCCAAGTGCAAAGGTGTCTG. GAPDH was used as an internal control. The primers for GAPDH were forward, ATGCCATCACTGCCACTCA, and reverse, CCTGCTTCACCACCTTCTTG. The quantification of the relative gene levels was calculated using the 2^−ΔΔCt^ method [22].

### 2.9 Statistics analysis

All the experiments were repeated at least three times, and the data were represented as mean  ±  standard deviation (SD). *p*-Value was calculated by either a two-tailed unpaired Student’s test or one-way analysis of variance followed by Tukeys’s *post hoc* test. Statistical analysis was carried out by GraphPad prism 7.0 version (GraphPad Software, CA, USA). *p* < 0.05 was considered statistically significant.

## 3 Results

### 3.1 Liver cirrhosis affected the intestinal morphological structure

**Figure 1:**
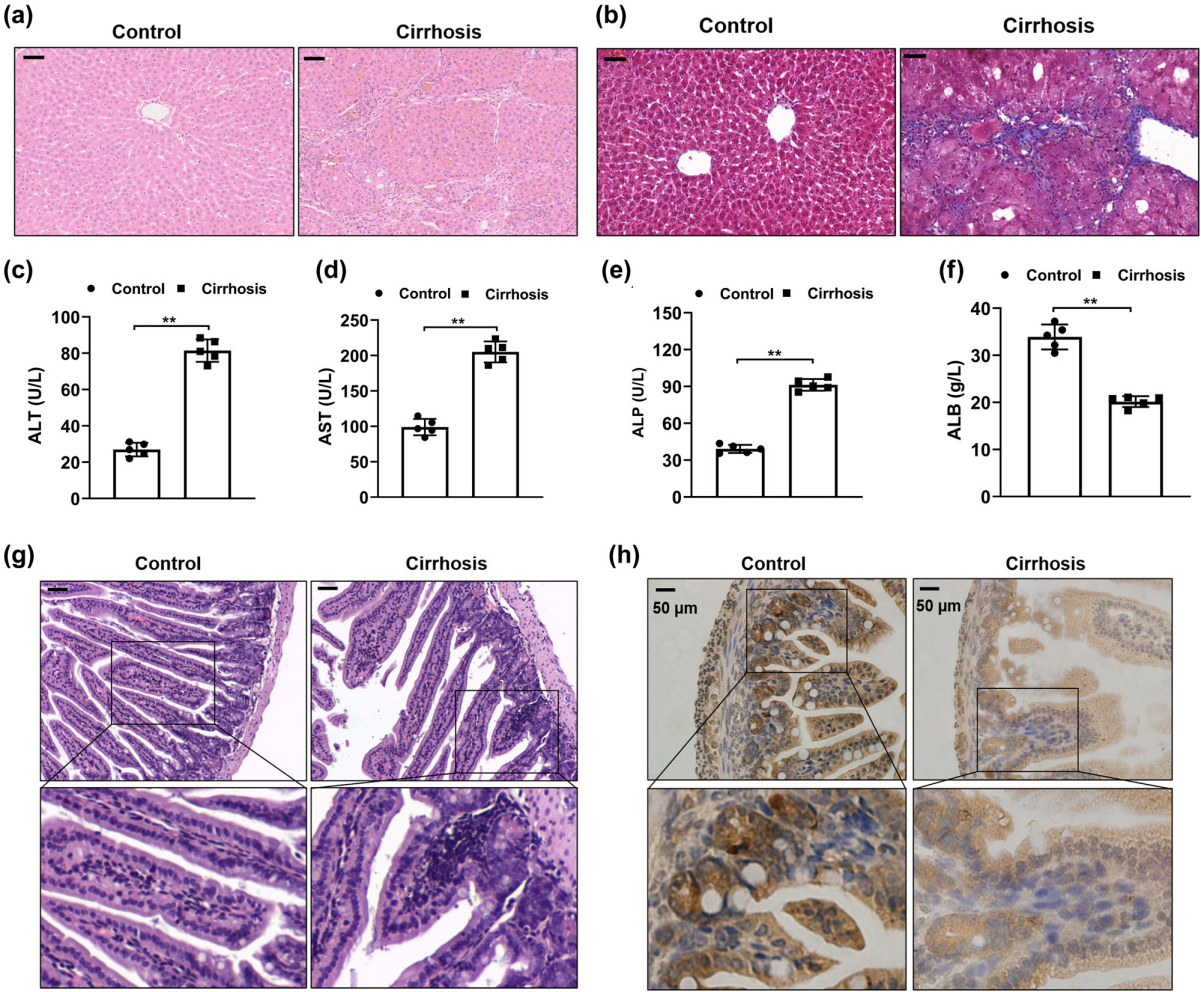
Liver cirrhosis affected the intestinal morphological structure. The rat liver cirrhosis model was established by CCl_4_ induction for 8 weeks. Histopathological characteristics in liver tissues with HE staining (a) and Masson Trichrome staining (b) (*n* = 5). Scale bar = 50 µm. Biochemical analysis of liver function between control and liver cirrhosis group: (c) ALT, (d) AST, (e) ALP, and (f) ALB (*n* = 5). (g) The histopathological changes in the intestines were evaluated by HE staining to investigate the impact of liver cirrhosis (*n* = 5). Scale bar = 50 µm. (h) Immunohistochemistry staining was applied to determine the expression of ZO-1. **p < 0.01 in 2-tailed *t*-test. Data are presented as mean ± SD.

We established a liver cirrhosis rat model using CCl_4_ treatment for 8 weeks. The morphological changes were detected through HE and Masson staining. As shown in Figure 1a and b, the results showed that in the control group, the hepatic lobule structure was clear, and hepatic cords were arranged radially around the central veins. However, the liver samples of the cirrhosis group represented the destroyed hepatic lobular structure and remarkable interstitial collagen deposition. Moreover, the data of serum biochemical indicators in rats showed that the levels of ALT, AST, and ALP in rat serum were remarkably increased in the cirrhosis group while the ALB level was lowered compared with the control group (Figure 1c–f). The results indicated that liver cirrhosis rat models were constructed successfully. We next sought to investigate the influence of liver cirrhosis on the structure of intestines using HE staining. As shown in Figure 1g, the control group had normal intestinal morphology. However, the liver cirrhosis group indicated severe intestinal mucosal damage with wider villi spacing and inflammatory cell infiltrations. Furthermore, the result of ZO-1 staining showed a significantly decreased expression of ZO-1 in intestinal tissues of the cirrhosis group compared to the control group (Figure 1h). The results demonstrated that liver cirrhosis triggered gut injury and intestinal barrier disruption.

### 3.2 Liver cirrhosis reduced 25(OH)-VD and decreased the levels of DEFA5 in intestinal tissues

**Figure 2:**
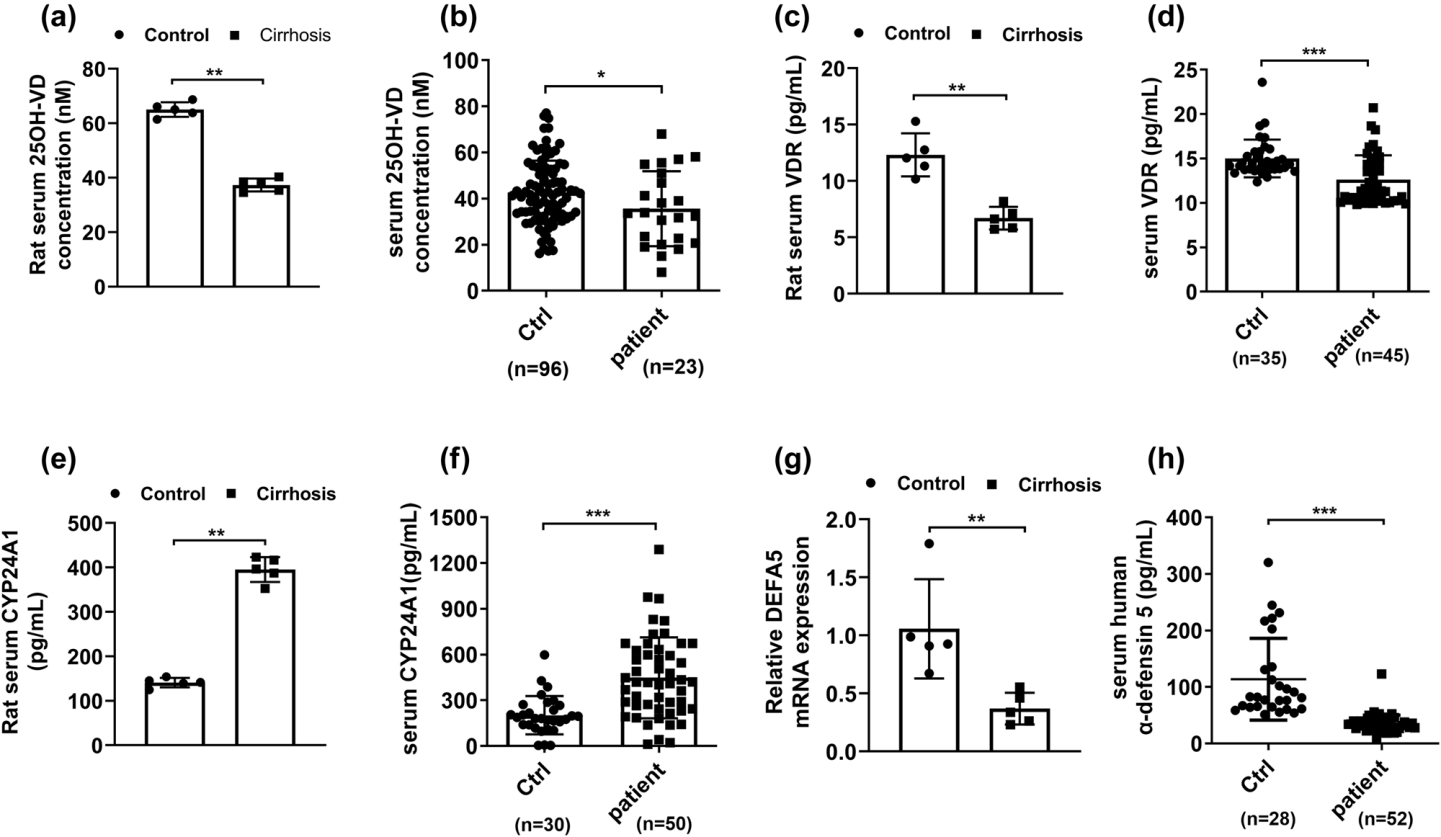
Liver cirrhosis reduced 25(OH)-VD and decreased the levels of DEFA5 in intestinal tissues. The levels of 25(OH)-VD (a and b), VDR (c and d), and 24-hydroxylation by mitochondrial 24-hydroxylase (CYP24A1) (e and f) of rat and human serum of liver cirrhosis were measured through ELISA. (g and h) The concentrations of defensin 5 (DEFA5) in rat intestinal tissues and human serum of liver cirrhosis were detected using RT-PCR and ELISA, respectively. *p < 0.05, **p < 0.01, ***p < 0.001 in 2-tailed *t*-test. Data are presented as mean ± SD.

The intestinal epithelium is the main target tissue of the biologically active form of vitamin D-25(OH)-VD_3_, which regulates intestinal absorption of calcium, phosphate, and other biological effects [23]. 25(OH)-VD_3_ predominantly acts through the VDR, a nuclear, ligand-dependent transcription factor. 25(OH)-VD_3_ is inactivated through 24-hydroxylation by mitochondrial 24-hydroxylase (CYP24A1) to lose its normal physiological functions [24]. We measured the levels of 25(OH)-VD_3_, VDR, and CYP24A1 in the ileum of rats and the serum of liver cirrhosis patients. As shown in Figure 2a–f, the concentrations of 25(OH)-VD_3_ (Figure 2a and b) and VDR (Figure 2c and d) were remarkably attenuated in rat and human serum of liver cirrhosis, and in contrast, augmented levels of CYP24A1 (Figure 2e and f) were detected compared with the control group. Enteric defensins are antibacterial peptides secreted by Paneth cells in the small intestine. We further examined the levels of defensin 5 (DEFA5) in rat intestinal tissues using RT-PCR analysis and in human serum of liver cirrhosis using enzyme-linked immunosorbent assay (ELISA). The results revealed that a down-regulation of DEFA5 was determined in rat intestinal tissues (Figure 2g) and human serum of liver cirrhosis (Figure 2h). To summarize, we hypothesized that liver cirrhosis reduced hepatic 25(OH)-VD and inhibited the levels of DEFA5 in intestinal tissues.

### 3.3 Vitamin D inhibited liver cirrhosis-induced intestinal pathological injury and inflammation

**Figure 3:**
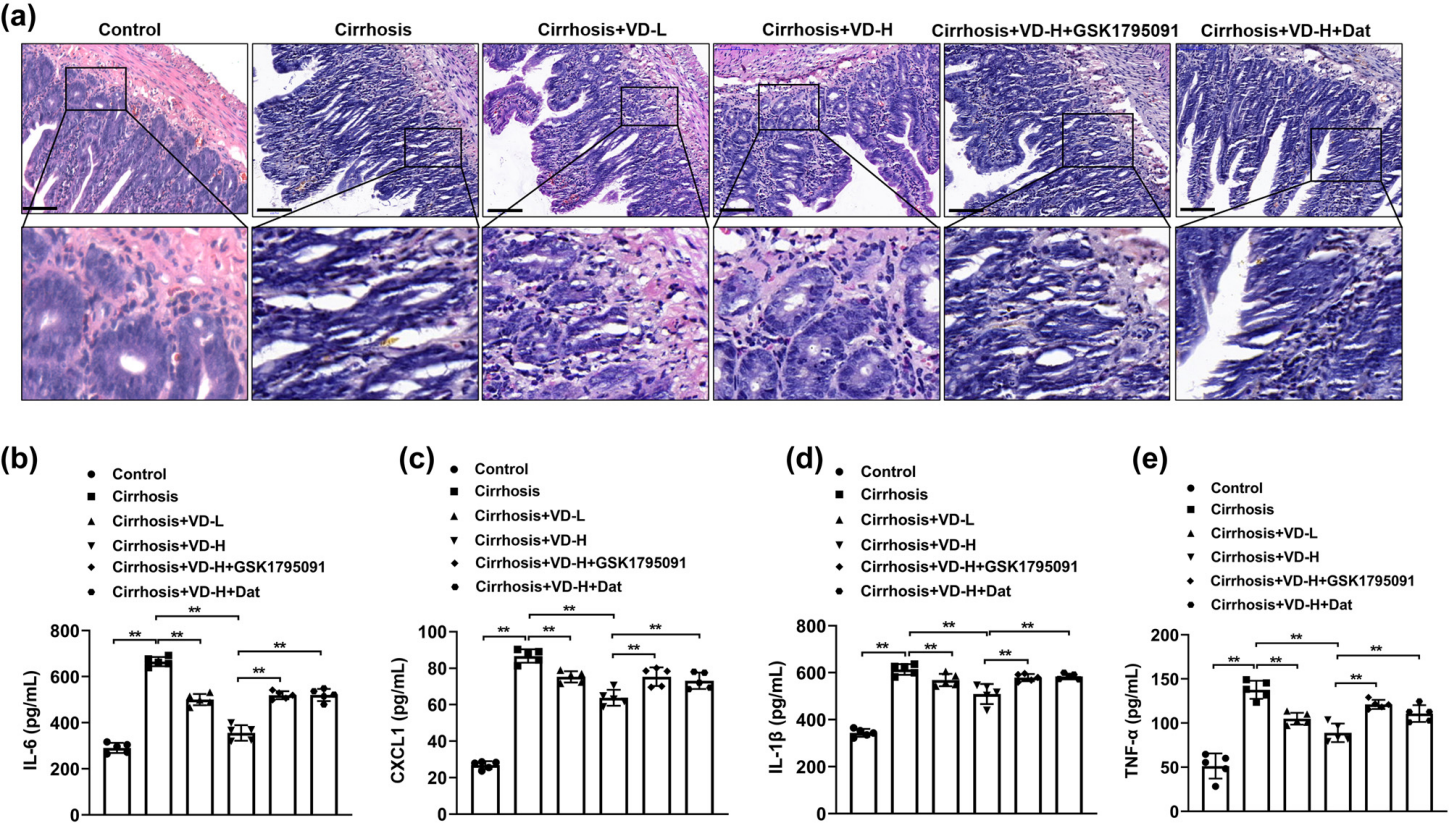
Vitamin D inhibited liver cirrhosis-induced intestinal pathological injury and inflammation. Vitamin D was intraperitoneally administered daily to 100 μL of vitamin D solution at different doses. The ileum tissues of each group were harvested for HE staining (a). *n* = 5, scale bar = 50 µm. VD-L: low-dose vitamin D group (500 IU/kg); VD-H: high-dose vitamin D group (2,000 IU/kg). The levels of inflammatory cytokines including IL-6 (b), IL-8 (c), IL-1β (d), and TNF-α (e) in the intestinal samples were determined by ELISA (*n* = 5). *p < 0.05, **p < 0.01 in Tukey’s *post hoc* comparisons test. Data are presented as mean ± SD.

Based on the above results, we concluded that liver cirrhosis caused severe injury and suppressed the concentrations of 25(OH)VD3 in intestinal tissues. Vitamin D acts a crucial role in safeguarding the intestinal barrier and preventing gastrointestinal mucosal inflammation. Ileum tissues of each group were stained with HE and the results are shown in Figure 3a. The intestinal injury was observed in liver cirrhosis rats with inflammatory cell infiltration and wider villi spacing compared to the control group. Rats with low-dose or high-dose vitamin D administration exhibited improved intestinal integrity. However, the treatment of GSK1795091 and Dat effectively reversed the vitamin D-mediated intestinal repair. Moreover, we next measured the levels of the inflammatory cytokines, including IL-6, CXCL1, IL-1β, and TNF-α, in the intestine to examine the role of liver cirrhosis on inflammatory response following vitamin D treatment. As shown in Figure 3b–e, the intestinal secretion levels of IL-6. CXCL1, IL-1β, and TNF-α were obviously increased in the liver cirrhosis group compared with the control group. And the treatment with low-dose or high-dose vitamin D significantly reduced inflammatory cytokine levels in the ileum tissues. Furthermore, the use of GSK1795091 and Dat obviously reversed the vitamin D-mediated inhibited inflammatory response. Hence, the intervention of vitamin D remarkably attenuated the secretion of inflammatory cytokines induced by liver cirrhosis.

### 3.4 Vitamin D treatment effectively suppressed oxidative stress activated by liver cirrhosis

**Figure 4:**
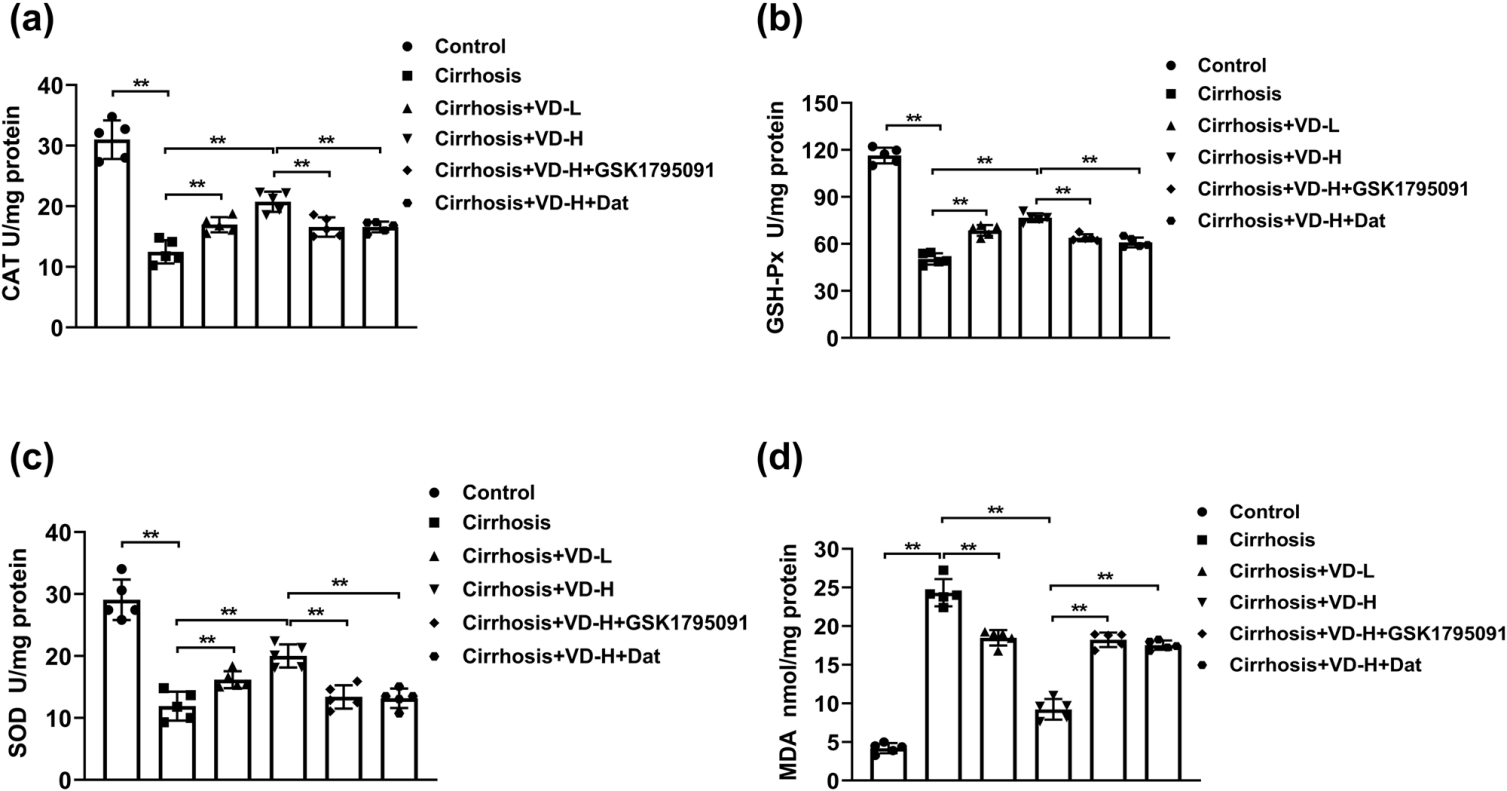
Vitamin D treatment effectively suppressed oxidative stress activated by liver cirrhosis. The levels of oxidative stress factors including CAT (a), GSH-Px (b), SOD (c), and MDA (d) in intestinal tissues were evaluated using ELISA kits (*n* = 5). *p < 0.05, **p < 0.01 in Tukey’s *post hoc* comparisons test. Data are presented as mean ± SD.

It has been reported that the regulation of oxidative stress plays crucial roles in the maintenance of intestinal homeostasis [25]. The changes in oxidative stress levels influence the microbial environment and the intestinal barrier integrity [26]. In our study, the oxidative damage was evaluated through the levels of CAT, GSH-Px, SOD, and MDA in intestinal tissues using ELISA kits. As shown in Figure 4a–d, the liver cirrhosis resulted in oxidative stress in the ileum tissues, reducing CAT, GSH-Px, and SOD and promoting MDA concentrations compared with the control group. In addition, the treatment of low-dose or high-dose vitamin D effectively inhibited oxidative stress by increasing CAT, GSH-Px, and SOD and attenuating MDA levels compared with the cirrhosis model group. However, the treatment of GSK1795091 and Dat effectively reversed the vitamin D-regulated oxidative stress. Taken together, liver cirrhosis may lead to oxidative stress in intestinal and the treatment of vitamin D significantly protects intestinal from oxidative stress-induced injury.

### 3.5 The intervention of vitamin D inhibited TLR4/MyD88/NF-κB signaling in the intestinal tissues of rats

**Figure 5:**
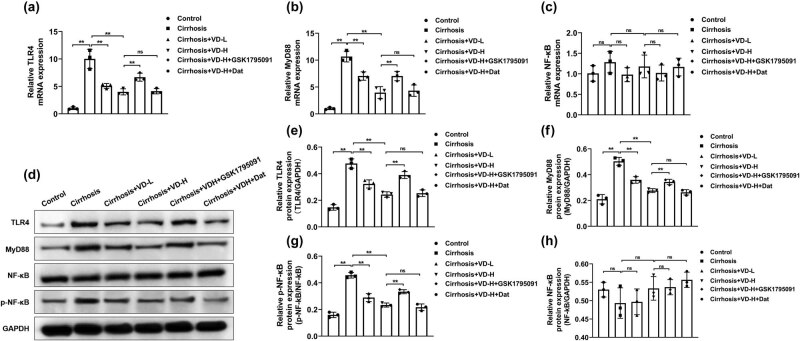
The intervention of vitamin D inhibited TLR4/MyD88/NF-κB signaling in the intestinal tissues of rats. (a–c) The mRNA expression of TLR4, MyD88, and NF-κB was validated using RT-PCR analysis (*n* = 3). GAPDH mRNA was used to normalize the relative gene expression. (d–h) Western blot analysis was performed to determine the protein levels of TLR4, MyD88, NF-κB, and p-NF-κB of rat intestinal samples (*n* = 3). GAPDH protein was used as the internal reference. ns, no significant difference, **p < 0.01 in Tukey’s *post hoc* comparisons test. Data are presented as mean ± SD.

The role of the TLR4/MyD88/NF-κB signaling pathway in mediating oxidative stress and inflammatory responses has been reported previously [27]. Given the role of TLR4 activation and the downstream adapter MyD88 in activating the NF-κB signaling pathway, we investigated the influences of liver cirrhosis on TLR4/MyD88/NF-κB signaling in ileum tissues using RT-PCR and Western blot analysis. As shown in Figure 5a–h, the results showed that liver cirrhosis activated TLR4/MyD88/NF-κB signaling in ileum samples of rats compared with the control group. Conversely, rats received low-dose or high-dose treatment of vitamin D exhibited decreased expression levels of TLR4, MyD88, and phospho-NF-κB p65 (p-NF-κB) in the ileum tissues compared with the liver cirrhosis model group. The treatment of GSK1795091 effectively reversed the vitamin D-mediated suppression of the TLR4/MyD88/NF-κB pathway while Dat had no significant influence on this. These findings unveiled that vitamin D suppressed the TLR4/MyD88/NF-κB signaling pathway activated by liver cirrhosis to facilitate intestinal damage repair.

## 4 Discussion

In the present study, we demonstrated that liver cirrhosis caused the intestinal damage. The expressions of 25(OH)-D_3_ and VDR decreased while that of 1,25-dihydroxyvitamin D [3] 24-hydroxylase (CYP24A1) was increased in intestinal tissues of liver cirrhosis rats. Moreover, liver cirrhosis inhibited the levels of DEFA5 in either cirrhosis rats or clinical patients’ serum. The treatment of vitamin D effectively promoted the repair of intestinal injury by inhibiting the oxidative stress and inflammation. Mechanistically, vitamin D might promote the process of liver cirrhosis-induced intestinal damage repair by suppressing the TLR4/MyD88/NF-κB signaling pathway. Taken together, the results indicated that vitamin D intervention provides novel insight into the potential therapy of liver cirrhosis-induced intestinal damage.

Evidences have revealed that liver cirrhosis is associated with intestinal structures and functions, including the epithelial barrier dysfunction, the increased intestinal permeability, and inflammatory infiltration [28]. Induction of CD14 + Trem-1 + iNOS + intestinal macrophages in liver cirrhosis patients released IL-6, NO, and accelerated intestinal permeability [6]. Regulation of oxidative stress is crucial for the maintenance of intestinal homeostasis [29]. The alcohol, intestinal microbiota alterations, and intestinal inflammation of patients with liver cirrhosis may induce gut oxidative damage [5]. Moreover, systemic inflammation and oxidative stress factors induced in the liver could be transferred to the intestines through blood [30]. Previous researches reported that liver cirrhosis could cause significant intestinal oxidative stress and the enzyme xanthine oxidase in the mucosa and enterocyte mitochondria was the important source of free radicals [31]. Decompensated liver cirrhosis was related to the activation of intestinal oxidative stress, potentially resulting in intestinal damage and higher levels of systemic endotoxemia in the patients [32].

As generally known, vitamin D acts crucial roles not only in affecting intestinal epithelial integrity but also in regulating gut inflammatory responses and innate immune barrier functions [33,34]. Several studies have reported that vitamin D is involved in the progression of inflammatory bowel diseases (IBD), including Crohn’s disease (CD) and ulcerative colitis (UC) [35]. Vitamin D acts via the VDR to mediate gene transcription. Previous researches indicated that vitamin D suppressed Th17 and Th1 responses, increased Tregs, and promoted antimicrobial defensins release, which is mainly secreted by Paneth cells [12]. Moreover, the association between vitamin D and intestinal functions in cirrhosis has attracted the attention of numerous researchers. Wang et al. [36] found that vitamin D enhances gut barrier integrity in cirrhosis rats by increasing the heme oxygenase-1 expression. Active vitamin D3 treatment attenuated intestinal permeability, inhibited bacterial translocation, and enriched beneficial gut microbiota in cirrhosis rats [37]. In addition, it had been revealed that vitamin D levels were associated with liver cirrhosis severity and patients’ mortality [38].

Toll-like receptors (TLRs) are transmembrane protein family receptors that play a key role in nonspecific or innate immune defense [39,40]. TLR4 is a key element in the TLR family. Previous reports revealed that the organism activated the innate immune responses after external stimulation and increased TLR4 level and triggered NF-κB through the MyD88-dependent pathway, which resulted in severe intestinal inflammation [41]. Furthermore, recent researches unveiled that the TLR4/MyD88/NF-κB signaling pathway was involved in the regulation of oxidative stress injury [42]. *Bacillus coagulans* TL3 regulated the TLR4/MyD88/NF-κB and Nrf2 signaling pathways in the cecal tissue of rats and regulated intestinal microflora to protect the intestine from inflammation and oxidative damage caused by LPS [43]. In this study, we found the activation of TLR4/MyD88/NF-κB pathway in intestinal tissues of liver cirrhosis rats while the treatment of vitamin D remarkably suppressed the TLR4/MyD88/NF-κB pathway.

There are shortcomings in this study. A limitation of this work was the small sample size of animal experiments due to the limited time and financial support. In future research, we may investigate the changes in the intestinal flora composition induced by liver cirrhosis to further confirm the findings. Besides, we focused on the influences of liver cirrhosis on the intestines in this study. The livers treated with vitamin D are our further research direction and we think that there may be some interesting findings.

In conclusion, we demonstrated that vitamin D negatively regulated liver cirrhosis-induced intestinal inflammation and oxidative stress through the TLR4/MyD88/NF-κB pathway, which revealed the practical implication for vitamin D supplementation as a strategy against cirrhosis-induced intestinal injury.

## References

[1] Yeom SK, Lee CH, Cha SH, Park CM. Prediction of liver cirrhosis, using diagnostic imaging tools. World J Hepatol. 2015;7(17):2069–79. 10.4254/wjh.v7.i17.2069. PMC4539400 26301049

[2] Garcia-Tsao G, Friedman S, Iredale J, Pinzani M. Now there are many (stages) where before there was one: In search of a pathophysiological classification of cirrhosis. Hepatology. 2010;51(4):1445–9. 10.1002/hep.23478. PMC2882065 20077563

[3] Trebicka J, Fernandez J, Papp M, Caraceni P, Laleman W, Gambino C, et al. The predict study uncovers three clinical courses of acutely decompensated cirrhosis that have distinct pathophysiology. J Hepatol. 2020;73(4):842–54. 10.1016/j.jhep.2020.06.013. 32673741

[4] Twu Y-C, Lee T-S, Lin Y-L, Hsu S-M, Wang Y-H, Liao C-Y, et al. Niemann-pick type C2 protein mediates hepatic stellate cells activation by regulating free cholesterol accumulation. Int J Mol Sci. 2016;17(7):1122. 10.3390/ijms17071122. PMC4964497 27420058

[5] Assimakopoulos SF, Tsamandas AC, Tsiaoussis GI, Karatza E, Zisimopoulos D, Maroulis I, et al. Intestinal mucosal proliferation, apoptosis and oxidative stress in patients with liver cirrhosis. Ann Hepatol. 2013;12(2):301–7. 10.1016/S1665-2681(19)31369-9. 23396742

[6] Du Plessis J, Vanheel H, Janssen CEI, Roos L, Slavik T, Stivaktas PI, et al. Activated intestinal macrophages in patients with cirrhosis release NO and IL-6 that may disrupt intestinal barrier function. J Hepatol. 2013;58(6):1125–32. 10.1016/j.jhep.2013.01.038. 23402745

[7] Albillos A, de Gottardi A, Rescigno M. The gut-liver axis in liver disease: Pathophysiological basis for therapy. J Hepatol. 2020;72(3):558–77. 10.1016/j.jhep.2019.10.003. 31622696

[8] Tripathi A, Debelius J, Brenner DA, Karin M, Loomba R, Schnabl B, et al. The gut–liver axis and the intersection with the microbiome. Nat Rev Gastroenterol Hepatol. 2018;15(7):397–411. 10.1038/s41575-018-0011-z. PMC6319369 29748586

[9] Stacchiotti V, Rezzi S, Eggersdorfer M, Galli F. Metabolic and functional interplay between gut microbiota and fat-soluble vitamins. Crit Rev Food Sci Nutr. 2021;61(19):3211–32. 10.1080/10408398.2020.1793728. 32715724

[10] Su D, Nie Y, Zhu A, Chen Z, Wu P, Zhang L, et al. Vitamin D signaling through induction of paneth cell defensins maintains gut microbiota and improves metabolic disorders and hepatic steatosis in animal models. Front Physiol. 2016;7:498. 10.3389/fphys.2016.00498. PMC5108805 27895587

[11] Bancil AS, Poullis A. The Role of Vitamin D in Inflammatory Bowel Disease. Healthc (Basel). 2015;3(2):338–50. 10.3390/healthcare3020338. PMC4939537 27417766

[12] Malaguarnera L. Vitamin D and microbiota: Two sides of the same coin in the immunomodulatory aspects. Int Immunopharmacol. 2020;79:106112. 10.1016/j.intimp.2019.106112. 31877495

[13] Merriman KE, Kweh MF, Powell JL, Lippolis JD, Nelson CD. Multiple β-defensin genes are upregulated by the vitamin D pathway in cattle. J Steroid Biochem Mol Biol. 2015;154:120–9. 10.1016/j.jsbmb.2015.08.002. 26255277

[14] Zhu Q, He G, Wang J, Wang Y, Chen W, Guo T. Down-regulation of toll-like receptor 4 alleviates intestinal ischemia reperfusion injury and acute lung injury in mice. Oncotarget. 2017;8(8):13678–89. 10.18632/oncotarget.14624. PMC5355129 28099145

[15] Moses T, Wagner L, Fleming SD. TLR4-mediated Cox-2 expression increases intestinal ischemia/reperfusion-induced damage. J Leukoc Biol. 2009;86(4):971–80. 10.1189/jlb.0708396. PMC2752016 19564573

[16] Cho SX, Rudloff I, Lao JC, Pang MA, Goldberg R, Bui CB, et al. Characterization of the pathoimmunology of necrotizing enterocolitis reveals novel therapeutic opportunities. Nat Commun. 2020;11:5794. 10.1038/s41467-020-19400-w. PMC7666196 33188181

[17] Giusto M, Barberi L, Di Sario F, Rizzuto E, Nicoletti C, Ascenzi F, et al. Skeletal muscle myopenia in mice model of bile duct ligation and carbon tetrachloride-induced liver cirrhosis. Physiol Rep. 2017;5(7):e13153. 10.14814/phy2.13153. PMC5392502 28364027

[18] Vuillermot S, Luan W, Meyer U, Eyles D. Vitamin D treatment during pregnancy prevents autism-related phenotypes in a mouse model of maternal immune activation. Mol Autism. 2017;8:9. 10.1186/s13229-017-0125-0. PMC5351212 28316773

[19] Tan X, Gao L, Cai X, Zhang M, Huang D, Dang D, et al. Vitamin D3 alleviates cognitive impairment through regulating inflammatory stress in db/db mice. Food Sci Nutr. 2021;9(9):4803–14. 10.1002/fsn3.2397. PMC8441317 34531993

[20] Castillo EC, Hernandez-Cueto MA, Vega-Lopez MA, Lavalle C, Kouri JB, Ortiz-Navarrete V. Effects of Vitamin D Supplementation during the Induction and Progression of Osteoarthritis in a Rat Model. Evid Based Complement Altern Med. 2012;2012:156563. 10.1155/2012/156563. PMC3479853 23118784

[21] Luo Y, Tian G, Zhuang Z, Chen J, You N, Zhuo L, et al. Berberine prevents non-alcoholic steatohepatitis-derived hepatocellular carcinoma by inhibiting inflammation and angiogenesis in mice. Am J Transl Res. 2019;11(5):2668–82. PMC6556646 31217846

[22] Ish-Shalom S, Lichter A. Analysis of Fungal Gne Expression by Real Time Quantitative PCR. Methods Mol Biol. 2010;638:103–14. 10.1007/978-1-60761-611-5_7. 20238263

[23] Fleet JC, Schoch RD. Molecular mechanisms for regulation of intestinal calcium absorption by vitamin D and other factors. Crit Rev Clin Lab Sci. 2010;47(4):181–95. 10.3109/10408363.2010.536429. PMC3235806 21182397

[24] Xu Y, Hashizume T, Shuhart MC, Davis CL, Nelson WL, Sakaki T, et al. Intestinal and hepatic CYP3A4 catalyze hydroxylation of 1α,25-dihydroxyvitamin D(3): implications for drug-induced osteomalacia. Mol Pharmacol. 2006;69(1):56–65. 10.1124/mol.105.017392. 16207822

[25] Liu M, Sun T, Li N, Peng J, Fu D, Li W, et al. BRG1 attenuates colonic inflammation and tumorigenesis through autophagy-dependent oxidative stress sequestration. Nat Commun. 2019;10(1):4614. 10.1038/s41467-019-12573-z. PMC6787222 31601814

[26] Chen Y, Yang B, Ross RP, Jin Y, Stanton C, Zhao J, et al. Orally administered CLA ameliorates DSS-induced colitis in mice via intestinal barrier improvement, oxidative stress reduction, and inflammatory cytokine and gut microbiota modulation. J Agric Food Chem. 2019;67(48):13282–98. 10.1021/acs.jafc.9b05744. 31690068

[27] Bing X, Xuelei L, Wanwei D, Linlang L, Keyan C. EGCG maintains Th1/Th2 balance and mitigates ulcerative colitis induced by dextran sulfate sodium through TLR4/MyD88/NF-κB signaling pathway in rats. Can J Gastroenterol Hepatol. 2017;2017:3057268. 10.1155/2017/3057268. PMC5748319 29404307

[28] Pijls KE, Jonkers DM, Elamin EE, Masclee AA, Koek GH. Intestinal epithelial barrier function in liver cirrhosis: an extensive review of the literature. Liver Int. 2013;33(10):1457–69. 10.1111/liv.12271. 23879434

[29] Aceto GM, Catalano T, Curia MC. Molecular aspects of colorectal adenomas: the interplay among microenvironment, oxidative stress, and predisposition. BioMed Res Int. 2020;2020:1726309. 10.1155/2020/1726309. PMC7102468 32258104

[30] Natarajan SK, Ramamoorthy P, Thomas S, Basivireddy J, Kang G, Ramachandran A, et al. Intestinal mucosal alterations in rats with carbon tetrachloride-induced cirrhosis: Changes in glycosylation and luminal bacteria. Hepatology. 2006;43(4):837–46. 10.1002/hep.21097. 16557555

[31] Ramachandran A, Prabhu R, Thomas S, Reddy JB, Pulimood A, Balasubramanian KA. Intestinal mucosal alterations in experimental cirrhosis in the rat: Role of oxygen free radicals. Hepatology. 2002;35(3):622–9. 10.1053/jhep.2002.31656. 11870376

[32] Tsiaoussis GI, Assimakopoulos SF, Tsamandas AC, Triantos CK, Thomopoulos KC. Intestinal barrier dysfunction in cirrhosis: Current concepts in pathophysiology and clinical implications. World J Hepatol. 2015;7(17):2058–68. 10.4254/wjh.v7.i17.2058. PMC4539399 26301048

[33] Fakhoury HMA, Kvietys PR, AlKattan W, Anouti FA, Elahi MA, Karras SN, et al. Vitamin D and intestinal homeostasis: Barrier, microbiota, and immune modulation. J Steroid Bioche Mol Biol. 2020;200:105663. 10.1016/j.jsbmb.2020.105663. 32194242

[34] Yamamoto EA, Jørgensen TN. Relationships Between Vitamin D, Gut Microbiome, and Systemic Autoimmunity. Front Immunol. 2020;10:3141. 10.3389/fimmu.2019.03141. PMC6985452 32038645

[35] Battistini C, Ballan R, Herkenhoff ME, Saad SM, Sun J. Vitamin D modulates intestinal microbiota in inflammatory bowel diseases. Int J Mol Sci. 2020;22(1):362. 10.3390/ijms22010362. PMC7795229 33396382

[36] Wang PF, Yao DH, Hu YY, Li Y. Vitamin D improves intestinal barrier function in cirrhosis rats by upregulating heme oxygenase-1 expression. Biomol Ther. 2019;27(2):222–30. 10.4062/biomolther.2018.052. PMC6430230 30173501

[37] Lee PC, Hsieh YC, Huo TL, Yang UC, Lin CH, Li CP, et al. Active Vitamin D 3 treatment attenuated bacterial translocation via improving intestinal barriers in cirrhotic rats. Mol Nutr Food Res. 2021;65(3):e2000937. 10.1002/mnfr.202000937. 33258263

[38] Triantos C, Kalafateli M, Aggeletopoulou I, Diamantopoulou G, Spantidea PI, Michalaki M, et al. Vitamin D-related immunomodulation in patients with liver cirrhosis. Eur J Gastroenterol Hepatol. 2020;32(7):867–76. 10.1097/MEG.0000000000001597. 31789949

[39] López-López S, Romero de Ávila MJ, Hernández de León NC, Ruiz-Marcos F, Baladrón V, Nueda ML, et al. NOTCH4 Exhibits Anti-Inflammatory Activity in Activated Macrophages by Interfering With Interferon-γ and TLR4 Signaling. Front Immunol. 2021;12:734966. 10.3389/fimmu.2021.734966. PMC8671160 34925319

[40] De Nardo D. Toll-like receptors: Activation, signalling and transcriptional modulation. Cytokine. 2015;74(2):181–9. 10.1016/j.cyto.2015.02.025. 25846205

[41] Impellizzeri D, Fusco R, Genovese T, Cordaro M, D’Amico R, Trovato Salinaro A, et al. Coriolus versicolor downregulates TLR4/NF-κB signaling cascade in dinitrobenzenesulfonic acid-treated mice: A possible mechanism for the anti-colitis effect. Antioxidants. 2022;11(2):406. 10.3390/antiox11020406. PMC8869697 35204289

[42] Du Z, Ma Z, Lai S, Ding Q, Hu Z, Yang W, et al. Atractylenolide i ameliorates acetaminophen-induced acute liver injury via the TLR4/MAPKs/NF-κB signaling pathways. Front Pharmacol. 2022;13:797499. 10.3389/fphar.2022.797499. PMC8815859 35126160

[43] Wang Y, Lin J, Cheng Z, Wang T, Chen J, Long M. Bacillus coagulans TL3 inhibits LPS-induced caecum damage in rat by regulating the TLR4/MyD88/NF-κB and Nrf2 signal pathways and modulating intestinal microflora. Oxid Med Cell Longev. 2022;2022:5463290. 10.1155/2022/5463290. PMC8843965 35178157

